# Assessment of HCV-RNA test results and access rates to antiviral treatment in patients with anti-HCV positivity—a 10-year analysis

**DOI:** 10.55730/1300-0144.5546

**Published:** 2022-09-18

**Authors:** Emine TÜRKOĞLU YILMAZ, Zafer PARLAK, Hüseyin Şener BARUT

**Affiliations:** 1 Department of Infectious Disease and Clinical Microbiology, Faculty of Medicine, Tokat Gaziosmanpaşa University, Tokat, Turkey; 2 Department of Infectious Disease and Clinical Microbiology, İzmir Buca Seyfi Demirsoy Training and Research Hospital, İzmir, Turkey

**Keywords:** Hepatitis C virus, chronic hepatitis C, elimination, prevalence

## Abstract

**Background/aim:**

Currently, hepatitis C virus (HCV) infection can be cured by direct-acting antivirals (DAAs). In this study, we aimed to find the rate of viremia among patients with a positive anti-HCV test and the rate of antiviral treatment given to viremic patients. We also aimed to reach patients with anti-HCV positivity but not tested for HCV-RNA, and patients, who were diagnosed with HCV-RNA positivity but received no treatment.

**Materials and methods:**

In this study, individuals tested for anti-HCV in Tokat Gaziosmanpaşa University Research and Application Hospital in the period between January 2010 and January 2020 were reviewed retrospectively. Anti-HCV positive patients, who were not tested for HCV-RNA, and HCV-RNA positive patients, who did not receive treatment, were called for a follow-up visit in the outpatient clinic.

**Results:**

The prevalences of anti-HCV positivity and viremia among patients were 2.24% and 0.67%, respectively. A HCV-RNA test was ordered in 71.7% of the anti-HCV positive patients. Antiviral treatment was not given to 44.4% of the viremic patients. Of the patients, who were called for a follow-up visit in the outpatient clinic, 3.9% attended the visit. Of these patients, 0.8% were HCV-RNA positive and 0.7% received treatment.

**Conclusion:**

Although the rate of HCV-RNA testing was relatively high in patients with anti-HCV positivity, almost half of them did not receive treatment. We could reach only one-third of the patients, who were called for a follow-up visit, and only a few patients received treatment. Individuals with anti-HCV positivity should be referred to a specialist without delay and HCV-RNA testing should be performed immediately to achieve HCV elimination targets. The likelihood of difficulties in reaching patients later should be considered.

## 1. Introduction

Hepatitis C virus (HCV) infection is one of the most important causes of liver cirrhosis and hepatocellular cancer all over the world. Therefore, HCV infection is accepted as an important public health threat [1]. It is estimated that 58 million individuals are infected with HCV globally and this figure is estimated to be 434,000 (274,000–959,000) in Turkey. The mean prevalence of viremic cases is 0.8% (0.5%–1.7%) in Turkey^1^

HCV infection is often asymptomatic. Acute HCV infection becomes chronic at a rate of 70%–85%. The risk of liver cirrhosis within 20 years ranges from 15% to 30% in patients with chronic hepatitis C (CHC).^1^ Because the infection is often asymptomatic, the diagnosis may be delayed and patients may present with the end-stage liver disease years later. The diagnosis of CHC is often made incidentally at blood donation, prepregnancy testing, premarital or preoperative screening, or during routine follow-ups of hemodialysis patients [2].

Because of the high replication rate and the lack of a proofreading mechanism in the causative virus, effective vaccines are not currently available against HCV infection. Therefore, the control of the disease is based on the treatment of infected patients and the prevention of new transmissions. Fortunately, there have been revolutionary developments in the treatment of CHC over the last 10 years. With the introduction of direct-acting antivirals (DAAs), CHC has become curable at rates of >95%. The World Health Organization (WHO) introduced a strategy for HCV elimination in 2016, aiming to diagnose 90% of patients, treat 80% of patients, and achieve a 65% reduction in HCV-related mortality by the year 2030 [3]^2^. In line with this goal, the Turkey Viral Hepatitis Prevention and Control Program was introduced in Turkey in 2018.^3^ With this study, we aimed to contribute to HCV elimination targets. We aimed to find the rate of viremia among patients with positive anti-HCV tests and the rate of antiviral treatment administration to viremic patients. Moreover, we aimed to reach two groups of patients including those, who tested positive for anti-HCV antibodies but were not tested for HCV-RNA, and those, who were positive for HCV-RNA but received no treatment.

## 2. Materials and methods

This is a retrospective and single-center study, which was conducted in Tokat Gaziosmanpaşa University University Research and Application Hospital in the period between January 2010 and January 2020. Data were accessed through the hospital automation system. The study was approved by the local ethics committee (Clinical Research Ethics Committee of Tokat Gaziosmanpaşa University University, School of Medicine, 20-KAEK 258).

Anti-HCV test results obtained in Tokat Gaziosmanpaşa University University Research and Application Hospital in the period between January 2010 and January 2020 were reviewed retrospectively and reactive results were included in the study. Clinical departments were recorded, from which the tests were ordered revealing anti-HCV positivity. These departments were classified under the following five categories as the departments of emergency, internal medicine, surgery, infectious diseases, and gastroenterology. The frequency of HCV-RNA testing in anti-HCV positive patients, the prevalence of viremic patients, rate of starting antiviral treatment in viremic patients, treatment responses, and distribution of HCV genotypes were investigated.

Patients with anti-HCV positivity but not tested for HCV-RNA, patients with HCV-RNA positivity, who did not receive treatment, and patients, who relapsed, were recorded and contacted by phone. The phone numbers of these patients were retrieved from the hospital automation system. Unavailable patients were called at least twice. On the phone call, patients were informed about test results, and, if they were interested, it was explained that they could apply to the infectious diseases department for further examination and treatment. We ensured privacy when interviewing a patient. The spouse or first-degree relatives were not informed. If the patient died and a first-degree relative answered the phone, no detailed information was provided about the patient’s condition. However, such individuals were informed that they could apply to our outpatient clinic for testing for screening purposes to detect a potential infection.

Telephone conversations were carried out following the steps listed below:

1. Self-introduction of the caller.
2. The name and surname of the patient were confirmed.
3. The patient’s consent was obtained for the interview.
4. Patients were asked whether they were informed about the results of anti-HCV testing performed in our hospital.
5. It was explained to uninformed patients that they could attend a follow-up visit in our outpatient clinic to discuss results, undergo further examinations, and receive treatment.
6. Patients, who had already been informed about anti-HCV testing results, were asked whether additional tests were performed and if they received any treatment.
7. Patients, who moved to another city, were advised to apply to infectious diseases or gastroenterology specialists.

### 2.1. Statistical analysis

The Statistical Package for Social Sciences (SPSS) 22 (IBM Corp., Armonk, NY, USA) software was used for data analysis. The conformity of the variables to the normal distribution was examined using visual (histograms and probability graphs) and analytical methods (Kolmogorov-Smirnov and Shapiro-Wilk tests). For descriptive statistics, numbers and percentages were used for categorical variables, mean ± standard deviation was used for normally distributed continuous variables, and median (minimum-maximum) was used for nonnormally distributed continuous variables.

## 3. Results

Anti-HCV tests performed in Tokat Gaziosmanpaşa University University Research and Application Hospital in the period between January 2010 and January 2020 were reviewed. When repeat tests for individual patients were excluded, we found that anti-HCV tests were performed on 132,215 patients, and anti-HCV positivity was revealed in 3249 of these patients. Anti-HCV prevalence was 2.24%. Of the patients, 62.6% (n = 2038) were women and the mean age was 56.38 ± 18.3 years.

**Figure 1:**
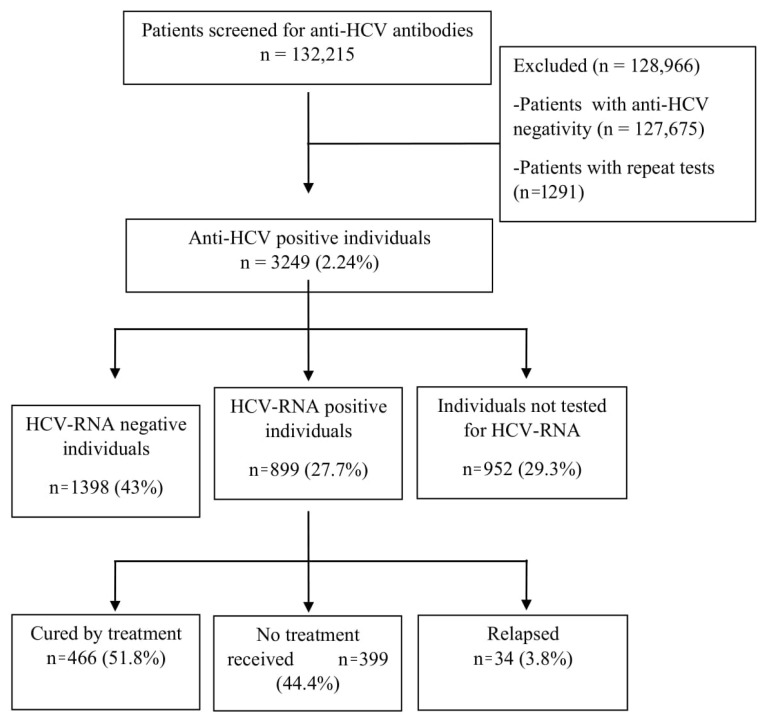
Distribution of anti-HCV positive patients by HCV-RNA test results and antiviral treatment status.

HCV-RNA testing was ordered for 2297 (70.7%) of the anti-HCV positive patients. In the period after 2016, when second-generation DAAs became available in our country, the rate of HCV-RNA testing was found to be 57.7% (424/734) in patients with anti-HCV positivity. HCV-RNA positivity was detected in 899 (27.7%) of all anti-HCV positive patients. The prevalence of viremic patients was 0.67 (899/132,215). Of the viremic patients, 65.7% (n = 591) were women and the mean age was 61.44 ± 12.66 years. Antiviral treatment was not started in 399 (44.4%) of the viremic patients. Of 500 patients treated, 466 (51.8%) were cured, and 34 (3.8%) relapsed (Figure 1).

The distribution of patients with anti-HCV positivity but with no HCV-RNA testing by clinical departments was shown in Table 1. It was found that 85.4% (n = 813) of the patients without HCV-RNA testing had not been referred to infectious diseases and/or gastroenterology clinics.

Because HCV genotyping was required for the selection and determining the duration of antiviral therapy during the period of the study, the rates of genotype testing were also investigated. Genotyping was studied in 62.6% (n = 563) of 899 viremic patients. The HCV genotypes of patients are presented in Table 2.

**Figure 2:**
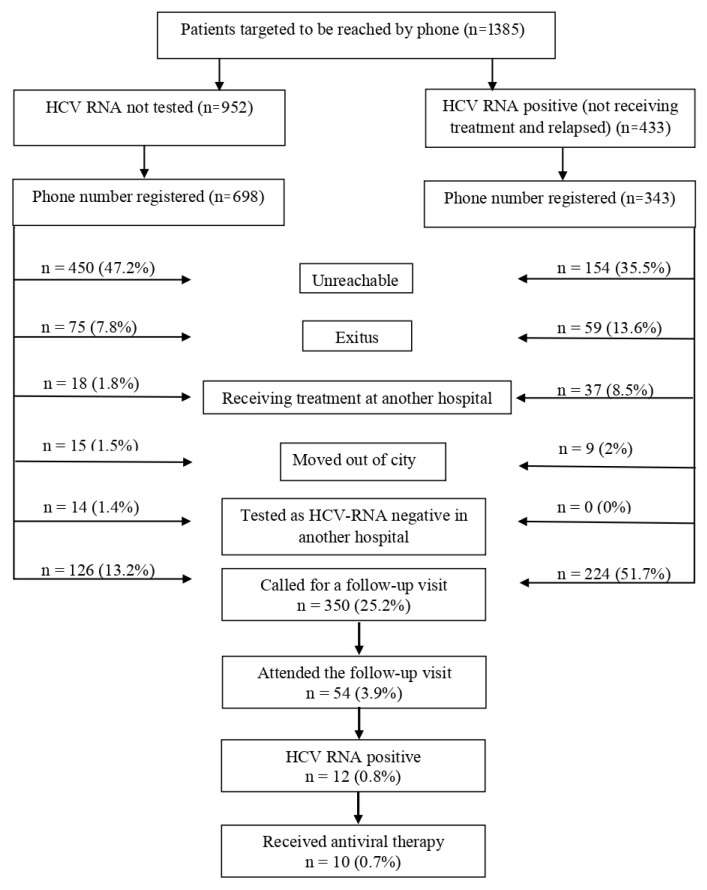
Outcome in anti-HCV positive patients, who were not tested for HCV-RNA, and in HCV-RNA positive patients, who did not receive treatment or relapsed.

The telephone numbers of 73.3% (n = 698) of the 952 patients not tested for HCV-RNA were available in the hospital automation system. Although all patients were called twice, 47.2% (n = 450) could not be reached. The telephone numbers of 79.2% (n = 343) of the 433 HCV-RNA positive patients (399 untreated, 34 relapsed) were available in the system. In this group of patients, 35.5% (n = 154) did not answer the call. The following reasons were noted for unavailable patients, including patients’ not answering the phone, the phone being turned off, busy signals, and incorrect and invalid phone numbers. Finally, only 54 (3.9%) of the 1385 patients, who were planned to be invited by a phone call for a follow-up visit at the hospital, attended visits to be tested or treated. HCV-RNA positivity was detected in 12 patients. Of these 12 patients, 10 were treated with DAA. Of the remaining two patients, one did not accept the treatment, and the other was a foreign national and treatment costs were neither affordable for this patient nor covered by the social security institution (Figure 2).

## 4. Discussion

Due to the COVID-19 pandemic, people with chronic diseases all over the world are at serious risk in terms of the odds of not only being infected with SARS-CoV-2 but experiencing poor disease management as well.^4^ Hepatitis C infection is acquired through parenteral exposure and CHC may develop while remaining asymptomatic in the majority of patients. However, CHC can cause cirrhosis and liver cancer if not diagnosed. Therefore, it is necessary to diagnose, treat, and regularly follow up on such patients. Because of such characteristics of disease management, CHC is among the chronic diseases most affected by the pandemic process [4]. As in other chronic diseases, significant problems are anticipated in the follow-up of CHC patients and a potential wave of liver-related morbidity and mortality during and after the pandemic is predicted. During the pandemic, significant disruptions have occurred in patients’ access to treatment and diagnostic approaches.^5^ In our study, we aimed to achieve cure in a significant number of patients but only 3.9% of the patients presented to the outpatient clinic and only 0.7% of the patients were treated.

After the introduction of HCV elimination targets in our country, similar studies to our study were published from many hospitals aiming to reach anti-HCV positive patients. While some of these studies included only patients, who were tested for anti-HCV antibodies preoperatively, others included all patients tested for anti-HCV for any purpose, as we did in our study. We reviewed serum test results of 132,215 patients and this is the highest number of patient serum samples tested, reviewed, and included in a study. The prevalence of anti-HCV positivity in the studies from Turkey ranges from 0.28% to 2.86% and the prevalence of viremic patients varies between 0.05% and 0.38% [2,5–8]. In our study, the prevalences of anti-HCV positivity (2.24%) and viremic patients (0.67%) were higher compared to similar studies from Turkey.

Anti-HCV antibody testing is the first-line test for the diagnosis of HCV infection. In the presence of a reactive anti-HCV antibody test result, demonstration of viremia is required to confirm active infection. For this purpose, the presence of HCV-RNA should be demonstrated by molecular methods. Despite persisting anti-HCV positivity, HCV-RNA becomes negative in patients with sustained virological response, resulting in spontaneous resolution or cure [9]. In our study, we found that HCV-RNA testing was performed in 71.7% of the patients with anti-HCV positivity. This rate is higher than those reported by similar studies [2,5–8]. The lack of HCV-RNA testing in approximately one-third of patients may indicate that clinicians did not follow up on test results after placing test orders. In our study, we also evaluated the frequency of HCV-RNA test orders for anti-HCV positive patients by clinics. We found that a significant portion (78.3%) of the patients admitted to the emergency department was not tested for HCV-RNA. Because an emergency department is an intense work environment, we think that patients may not have been informed about test results and such patients may not have been referred to relevant specialists. In the internal medicine and surgical clinics, approximately one-fourth of the patients were not tested for HCV-RNA. We think that this may be due to the lack of information. The lowest rate of the lack of HCV-RNA testing was in the infectious diseases department. We think that the reason for the failure to schedule further examinations in gastroenterology and/or infectious diseases clinics may have been unattended follow-up visits by patients for the discussion of test results.

Since CHC is a curable disease, antiviral treatment is very important to prevent not only complications but also disease transmission. The TURHEP study, which was published in 2015, reported that approximately 514,000 patients were infected with HCV in Turkey; 5500 of these patients were newly diagnosed, and only 4200 (0.8%) patients received antiviral treatment [10]. The low rate of access to antiviral treatment among patients infected with HCV is not unique to our country. In a study from the United Kingdom, it was reported that only 39% of people infected with HCV were referred to hepatology clinics, and 22% could receive treatment [11]. Studies from different countries have shown that 50%–80% of the patients infected with HCV were not diagnosed and less than 20% received antiviral treatment [12]. In our study, we found that 44.4% of viremic patients did not receive treatment. Moreover, when viremic patients, who were not tested for HCV-RNA, are included, this calculation will result in a lower figure than previously found. Because of the retrospective design of our study, we could not identify reasons for not starting treatment for viremic patients. We suggest that the reasons for failure to start treatment may include patients’ comorbidities, biopsy findings not meeting the treatment criteria relevant to the study period, the patient’s refusal of interferon treatment, and the patient’s failure to attend follow-up visits.

Genotype 1 is the most common worldwide, accounting for 46.2% of all HCV cases. Of those, who underwent subtyping, 31% are genotype 1a and 68% are genotype 1b. The second most common (30.1%) type is genotype 3. Genotypes 2, 4, and 6 constitute 22.8% of all cases, while the frequency of genotype 5 is less than 1% [13]. Genotype 1 is the most common subtype in studies from Turkey. Genotype 3 is the second most common subtype followed by genotypes 2 and 4. Subtype 1b constitutes the majority of genotype 1 cases [10,14]. Our results are similar to those reported in the literature.

The percentage of patients presenting to our hospital to be tested or treated was considerably lower than we expected. This result shows that patients should be informed about the positive test results immediately. Otherwise, it is difficult to reach such patients later. In 85.4% of the cases, HCV-RNA was not tested and these patients did not present to the infectious diseases or gastroenterology outpatient clinics. This appears to result from inadequate information delivery to patients by physicians of other specialties and the failure to refer patients to relevant clinics. A solution to this problem can be the creation of alert signals in hospital automation systems. For patients with positive anti-HCV test results, a specific alert sign may appear and remind the physician of referring this patient to the infectious diseases or gastroenterology outpatient clinic. Information sharing meetings about HCV can be organized to include physicians from all specialties and raise awareness.

In the study by Erbay et al., patients, who were found to be positive for anti-HCV antibodies during the preoperative screening procedure, were called by phone and asked whether they received information about test results. It was found that 29.8% of individuals, who were informed of test results, had never visited an infectious diseases/gastroenterology outpatient clinic. Moreover, 14.2% of individuals, who were newly diagnosed and informed by the surgeon, did not see a specialist [2]. In a study from the USA, anti-HCV and HCV-RNA positive individuals, who were diagnosed in the period between the years 2001 and 2008, were informed by a letter and a follow-up questionnaire was administered to those individuals 6 months later. It was reported that 50.3% of the respondents knew that they were infected with HCV, and 22.9% did not see a specialist [12]. In our study, we believe that the number of patients, who attended the follow-up visits after phone calls, was small not only because of the lack of awareness of HCV infection but also because of factors associated with the COVID-19 pandemic. One may suggest that patients would have liked to avoid waiting in a crowded hospital hall and catching the infection.

A major limitation of our study is its retrospective design. Because of the retrospective design of the study, we could not clarify why HCV-RNA positive individuals did not receive treatment. Another limitation was that the phone numbers of the patients were incomplete or incorrectly recorded in the hospital automation system. Therefore, a significant number of patients could not be reached.

## 5. Conclusion

It is difficult to access anti-HCV positive patients later. For this reason, patients with a positive test result should be informed timely and referred to infectious diseases or gastroenterology specialists without delay. Information can be provided at meetings with the participation of experts of several relevant specialties. Alerts can be created in the hospital automation system to notice physicians. Alerts can be in the form of buttons with a warning letter dedicated to directing anti-HCV positive patients to infectious diseases or gastroenterology specialists.

## Figures and Tables

**Table 1:** Distribution of nontesting for HCV-RNA among anti-HCV positive patients by clinical departments.

Departments	Nontesting for HCV-RNA/anti-HCV positivity (n)	Rates of nontesting for HCV-RNA (%)
Emergency department	47/60	78.3
Surgical outpatient clinics	383/1343	28.5
Internal medicine outpatient clinics	383/1431	26.7
Gastroenterology	98/923	10.3
Infectious diseases	38/783	4.8
Total	952/4540×	

× The figure is greater than the sum because testings were ordered by more than one clinic for individual patients.

**Table 2:** Genotype distribution.

Genotype	n	%
1× (unspecified subtype)	73	13
1b	439	78.1
1a	26	4.6
3	13	2.3
2	5	0.9
4	5	0.9
1a+1b	1	0.2
1a+3	1	0.2
Total	563	100

× Genotype subtyping was not performed for patients before the year 2013.

## Footnotes

1 World Health Organization (2021). Hepatitis C [online]. Website https://www.who.int/news-room/fact-sheets/detail/hepatitis-c [accessed 27 July 2021].

2 World Health Organization (2016). Global Health Sector Strategy on Viral Hepatitis 2016-2021. Towards Ending Viral Hepatitis [online]. Website https://www.who.int/publications/i/item/WHO-HIV-2016.06 [accessed 17 July 2021].

3 Ministry of Health, Turkey (2018). Turkey Viral Hepatitis Prevention and Control Program [online]. Website https://hsgm.saglik.gov.tr/depo/birimler/Bulasici-hastaliklar-db/duyurular/Turkiye_Viral_Hepatit_Onleme_ve_Kontrol_Programi/Turkiye_Viral_Hepatit_Onleme_ve_Kontrol_Programi_TR.pdf [accessed 17 July 2021].

4 Centers for Disease Control and Prevention (2021). What to Know About Liver Disease and COVID-19 [online]. Website https://www.cdc.gov/coronavirus/2019-ncov/need-extra-precautions/liver-disease.html [accessed 19 July 2021].

5 World Hepatitis Alliance (2020). Latest News. COVID-19 and Viral Hepatitis [online]. Website https://www.worldhepatitisalliance.org/missing-millions/covid-19/#covid-19-and-viral-hepatitis- [accessed 20 July 2021].
